# A New Industrial Technology for Mass Production of Graphene/PEBA Membranes for CO_2_/CH_4_ Selectivity with High Dispersion, Thermal and Mechanical Performance

**DOI:** 10.3390/polym12040831

**Published:** 2020-04-05

**Authors:** Samy Yousef, Zahid Sarwar, Justas Šereika, Nerijus Striūgas, Edvinas Krugly, Paulius Pavelas Danilovas, Dainius Martuzevicius

**Affiliations:** 1 Department of Production Engineering, Faculty of Mechanical Engineering and Design, Kaunas University of Technology, LT-51424 Kaunas, Lithuania; 2 Department of Materials Science, South Ural State University, Lenin Prospect 76, 454080 Chelyabinsk, Russia; 3 Faculty of Chemical Technology, Kaunas University of Technology, LT-50254 Kaunas, Lithuania; zahid.sarwar@ktu.edu (Z.S.); edvinas.krugly@ktu.lt (E.K.); paudani@ktu.lt (P.P.D.); dainius.martuzevicius@ktu.lt (D.M.); 4 Lithuanian Energy Institute, Laboratory of Heat Equipment Research and Testing, Breslaujos 3, LT-44403 Kaunas, Lithuania; Justas.Sereika@lei.lt; 5 Lithuanian Energy Institute, Laboratory of Combustion Processes, Breslaujos 3, LT-44403 Kaunas, Lithuania; Nerijus.Striugas@lei.lt

**Keywords:** polyether block amide, PEBA nanocomposite membranes, graphene, CO_2_/CH_4_ selectivity, natural gas, biogas

## Abstract

Polyether block amide (PEBA) nanocomposite membranes, including Graphene (GA)/PEBA membranes are considered to be a promising emerging technology for removing CO_2_ from natural gas and biogas. However, poor dispersion of GA in the produced membranes at industrial scale still forms the main barrier to commercialize. Within this frame, this research aims to develop a new industrial approach to produce GA/PEBA granules that could be used as a feedstock material for mass production of GA/PEBA membranes. The developed approach consists of three sequential phases. The first stage was concentrated on production of GA/PEBA granules using extrusion process (at 170–210 °C, depending on GA concentration) in the presence of Paraffin Liquid (PL) as an adhesive layer (between GA and PEBA) and assisted melting of PEBA. The second phase was devoted to production of GA/PEBA membranes using a solution casting method. The last phase was focused on evaluation of CO_2_/CH_4_ selectivity of the fabricated membranes at low and high temperatures (25 and 55 °C) at a constant feeding pressure (2 bar) using a test rig built especially for that purpose. The granules and membranes were prepared with different concentrations of GA in the range 0.05 to 0.5 wt.% and constant amount of PL (2 wt.%). Also, the morphology, physical, chemical, thermal, and mechanical behaviors of the synthesized membranes were analyzed with the help of SEM, TEM, XRD, FTIR, TGA-DTG, and universal testing machine. The results showed that incorporation of GA with PEBA using the developed approach resulted in significant improvements in dispersion, thermal, and mechanical properties (higher elasticity increased by ~10%). Also, ideal CO_2_/CH_4_ selectivity was improved by 29% at 25 °C and 32% at 55 °C.

## 1. Introduction

Natural gas is the cleanest and cheapest fossil fuel available at present. Its advantages made it a key player in the field of electricity generation and in majority of critical industrial sectors, including cements, iron and steel, etc. [1,2]. As demand for natural gas has been growing enormously recently, many energy-conversion technologies (e.g., Pyrolysis, fermentation, etc.) were employed to generate biogas from different types of waste in order to compensate the shortage in production of natural gas, and to dispose such waste simultaneously [3,4,5]. Usually, biogas obtained using such technologies contains many components, particularly 60–70 wt.% of Methane (CH_4_) as a main component, 30–40 wt.% of Carbon dioxide (CO_2_) as a significant impurity in natural gas paths, and some trace elements (e.g., Nitrogen, ammonia, hydrogen sulphide, water vapor, etc.) [6,7]. However, presence of CO_2_ in obtained biogas can result in some serious technical problems, for example, reduced transportation capacity, lower natural-gas heating value, and corrosion of pipeline [8]. Therefore, the biogas conversion process is usually followed by biogas upgrading and gas separation [9]. Currently, amine technology is widely used for Carbon absorption, however, this technology has several limitations, including large capital and operating costs, high footprint, etc., [10,11].

In order to avoid the limitations of amine technology, polymer membrane-based approach has been developed as an alternative and effective solution for CO_2_/CH_4_ gas separation [12]. These types of membranes are characterized by low cost, system compactness, process flexibility, less energy, simplicity during the operation and maintenance, good mechanical strength, and mastery to control thermodynamic solubility constraints [13]. Many examples can be found in literature, how polymer membranes have been utilized for gas separation, including Polyether block amide (PEBA), (PEO)-based polymers, perfluoropolymers, Pebax, PIMs, thermally rearranged, and iptycene-containing, etc. [14]. Among these polymer membranes, PEBA is classified as one of the most promising polymer membranes with high efficiency in CO_2_ separation due to its high CO_2_ permeability and CO_2_/CH_4_ selectivity [15,16]. In order to improve their permeability and selectivity and make them fulfil the industrial requirements and increase stability for the long term, PEBA mixed-matrix composite membranes (MCM) were developed to address these aspects through mixing or modifying PEBA by other additives or chemical treatments [17,18]. 

With regard to such membranes, many different types of filler materials (e.g., MOF-801 nanocrystal, TiO_2_ nanoparticles, Fe_2_O_3_ nanoparticles, nanozeolite, zeolite 13X nanoporous, carbon nanotubes, SiO_2_, Al_2_O_3_, etc.) were mixed or deposited onto a PEBA layer to create selective transport channels for gas separation [19,20,21,22,23,24,25,26,27]. Although many studies demonstrated that due to its unique properties, Graphene Oxide (GO) as a nanofiller has high CO_2_ selectively [28,29], but its potential application with PEBA in gas separation has not been analyzed yet. Also, GO is typically synthesized from graphite in the form of monolayer or few layers contains a lot of defects (crumpled) and oxygen (~30 wt.%) what affect negatively on the characterizations of graphene, including the selectively performance. In contrast, Graphene nanosheets (GA) have several layers of carbon up to 10–20 or even more but a very low level of oxygen content [30], therefore, GA was used in the present research. Only one study was conducted using graphene to produce PEBA membrane for pervaporation of butanol aqueous media [31]. Also, recently graphene-based membranes have been employed in other fields like fuel cells, anion exchange membranes, etc. [32,33,34]. Although polymer nanocomposite membranes have been widely studied on the lab scale, however, they have not been commercialized yet due to their poor dispersion and because these types of membranes are mostly challenged by nanofiller distribution and production of defect-free membranes with a very thin selective film [35,36]. Therefore, our research group used twin screw extruder as an industrial technique to mix graphene with PEBA and to produce graphene/PEBA filaments, then we spun into fibrous membranes using melt electrospinning for cleanup of oil spills [37]. Also, 2 wt.% of Paraffin Liquid (PL) was added during the extrusion process to create an adhesive layer on the surface of PEBA pellets and to facilitate incorporation of nanofillers on the pellet surface with uniform dispersion [38]. The results revealed that the developed dispersion technique can improve thermal and mechanical properties of the obtained nanocomposite. Based on this, the dispersion technique developed by our group was employed on GA/PEBA granules with uniform dispersion and to process GA/PEBA membranes using solution-casting method. CO_2_/CH_4_ selectivity, morphology, physical, chemical, thermal, and mechanical behaviors of the synthesized membranes were investigated by using SEM, TEM, XRD, FTIR, TGA-DTG, and universal testing machine. 

## 2. Experimental

### 2.1. Materials and Methodology

**Figure 1:**
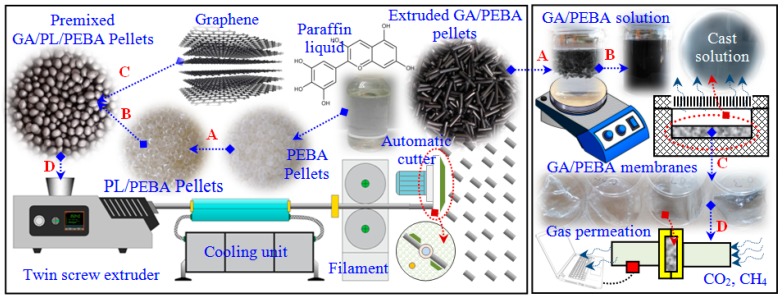
GA/PEBA membranes’ fabrication flowchart based on the developed approach.

Polyether block amide (PEBA) in the form of granules was supplied by Arkema Group, France (product No Pebax 3533SP-01). Paraffin Liquid (PL) of pharmaceutical grade was supplied by PanReac AppliChem, Germany (Prroduct No 146257). Graphene nanosheets (GA) of 10–20 nm thickness and a few micrometers in length were synthesized using multi-roll milling technique [39]. The present research started with the preparation of GA/PEBA granules using a premix process (mechanical mixer) followed by the main mixing process using a twin screw extruder. After that, solution-casting method with magnetic stirring were used to synthesize GA/PEBA membranes from the obtained GA/PEBA granules. All preparation steps are presented in Figure 1. 

### 2.2. Preparation of GA/PEBA Granules

**Figure 2:**
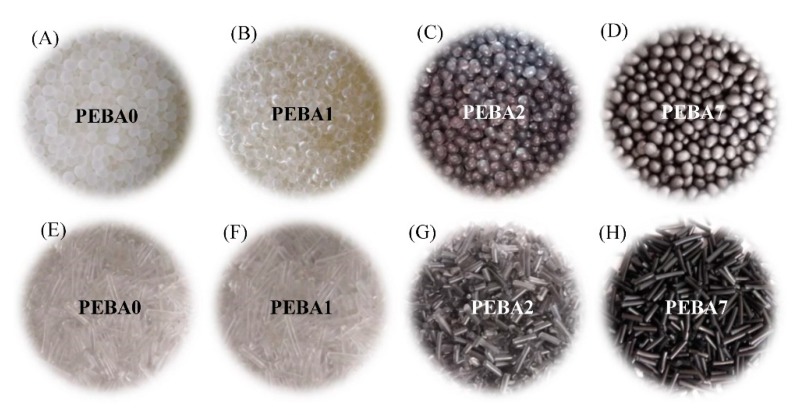
Images of (**A**–**D**) the pre-mixed GA/PEBA pellets and (**E**–**H**) the extruded GA/PEBA granules.

Two different types of mixing techniques were used at this stage to prepare GA/PEBA granules with uniform dispersion: premix and main mixing process. Both of them were selected based on the traditional production equipment of polymer products without needing for any special facilities or toxic chemicals. The premix process using a mechanical mixer in the presence of 0.2 wt.% of PL was used to establish a thin liquid film on the outer surface of PEBA pellets. When GA (with concentrations 0.05, 0.1, 0.2, 0.3, 0.4, and 0.5 wt.%) was added to PEBA pellets coated by PL, the thin liquid film converted to adhesive layer started to attract GA particles and distribute them uniformly on the pellet surfaces under the mixing conditions (Figure 2A–D). Then the second mixing process (main mixing process) using a twin screw extruder was employed to produce GA/PEBA granules by feeding the pre-mixed pellets into a hopper of a twin screw extruder with die diameter of 16 mm at feeding time of 2 min, die temperature of 160–220 °C (based on the GA concentration), mixing time of 4 min., and mixing speed of 30 rpm. The die was connected to the water cooling system and automatic rotating collector unit to obtain GA/PEBA granules with different concentration of GA and each batch was given a code based on PL and GA *w/w* percent particularly, 0PL/0GA “***PEBA0***”; 0.2PL/0GA “***PEBA1***”; 0.2PL/0.05GA “***PEBA2***”; 0.2PL/0.1GA “***PEBA3***”; 0.2PL/0.2GA “***PEBA4***”; 0.2PL/0.3GA “***PEBA5***”; 0.2PL/0.4GA “***PEBA6***”; 0.2PL/0.5GA “***PEBA7***”). Figure 2E–H shows the received virgin PEBA pellets and PEBA nanocomposite granules (at the lowest and the highest Graphene concentration, respectively) in the end of the extrusion process and after cutting by an automatic cutter. 

### 2.3. Preparation of GA/PEBA Membranes

**Figure 3:**
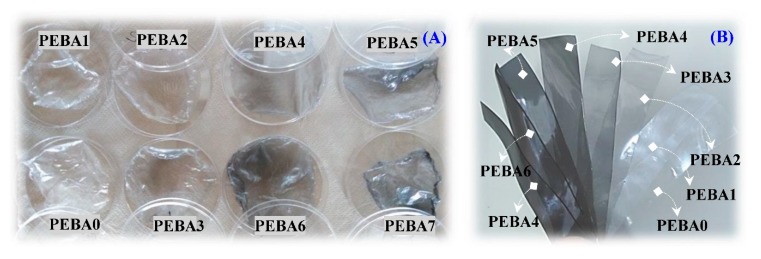
Images of (**A**) the synthesized GA/PEBA membranes and (**B**) the prepared GA/PEBA mechanical samples.

The solution-casting technique was utilized to fabricate GA/PEBA membranes from the obtained GA/PEBA granules at the end of the extrusion process. The initial experiments were performed to determine an appropriate liquid medium of GA/PEBA granules by using many different solvents, including concentrated and diluted ethanol, concentrated and diluted acetic acid, nitric acid, etc. The initial results showed that acetic acid and nitric acid can dissolve the extruded pellets. Since the acetic acid is classified as a green solvent and nitric acid as a toxic solvent, acetic acid was used in the main experiments to prepare GA/PEBA solutions with a solid to liquid ratio of 1 g (Pellets): 10 mL (Acetic Acid), applying magnetic stirring at 80 °C and 800 rpm for 30–40 min (based on the concentrations of GA) with a reflux condenser. The prepared solutions were poured individually into a Teflon mold with inner diameter of 90 mm, followed by solvent evaporation at room temperature for 4 h to avoid any thermal degradation in the obtained membranes. The dried thin films were peeled off from the Teflon die, then dried again in a vacuum oven for overnight at 25 °C to remove the residual solvent and to obtain membranes with a diameter of 90 mm and thickness in the range of 45–50 μm so that permeability test could be performed, as showing in Figure 3A. One more thick film was fabricated from each batch again with diameter 90 mm and thickness 200 μm for mechanical testing to be more stable during testing process. After that, manual cutter was employed to cut the mechanical tensile specimens (100 mm and width 10 mm) (Figure 3B).

### 2.4. Membrane Characterizations

Morphology and dispersion of GA in the fabricated membranes were examined using Scanning electron microscope (SEM) and Transmission electron microscopy (TEM) after having them coated with a gold layer. Chemical structures of the membranes were analyzed using the Fourier-Transform Infrared spectroscopy (FTIR, Vertex70 spectrometer) and X-ray crystallography (XRD). Thermal behaviors of the obtained membranes in terms of thermal stability, crystallinity degree, and melting temperature were determined by Thermogravimetric, Derivative-Thermogravimetric analysis (TGA-DTG), TA instruments TGA Q500 and Differential Scanning Calorimeter (DSC mod. Q-100 supplied by TA Instruments). The DSC measurement was conducted on 5–8 mg of each batch in the temperatures range of 50–900 °C at a heating rate of 10 °C per minute in nitrogen ambient; then the crystallinity degree of each batch according to Equation (1) was calculated [40]. Mechanical tensile properties of the membranes were measured by the Lloyd Universal Testing Machine (model LR10K) with rubber fixation jaws with loading rate of 200 ± 10 mm/min at ambient temperature.

(1) $$Xc\;(\%)=\frac{\Delta Hc}{\left(1-Ø\right)\Delta H_{m}^{o}}\;\times \;100$$

where Δ*Hc* is the apparent crystallization entropy of the tested filament and membrane samples, Δ$H_{m}^{o}$ is the melting enthalpy of 100% crystalline PEO and PA-6 which is equal to 166.4 J/g and 246 J/g respectively [41], while Ø represents the weight fraction of PL (0 and 0.2 wt.%) and GA (0, 0.05, 0.1, 0.2, 0.3, 0.4, and 0.5 wt.%) in the PEBA composites. Since PA represents the main fraction by mass, we focused on its crystallinity only during DSC measurements.

### 2.5. Setup of Gas Permeation and Membranes Holder

**Figure 4:**
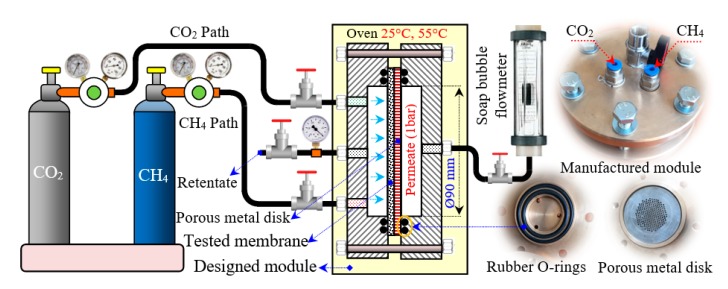
Scheme of the test rig used to perform gases permeation test.

Gas permeation of the synthesized membranes was evaluated using a test rig built especially for that purpose. The setup consisted of two separated CO_2_ and CH_4_ sources with a controlled flow rate, membrane holder, and gas measurements as shown in Figure 4. The permeability of the prepared membranes embedded in the membrane holder was measured by a constant pressure method. The holder was composed from two flanges made from stainless steel (grade 316), two rubber O-rings used as a pressure-tight seal between the polymeric membrane and the metal flanges with the effective membrane diameter of about 90 mm. In order to minimize possible errors due to membrane thickness and to avoid any deformation or destruction of membranes under the applied testing pressure and flow rate, the tested membrane surface was supported by porous metal disc with an outer diameter of 100 mm and 927 holes with a diameter of 1.5 mm. Small holes were distributed circularly and equally on the circumference of the disc’s surface. Also, the system had pressure regulators/transducer for adjusting of pressure during experiments and control valves/flow meter for adjusting of gas volumetric flow rate. 

Gas permeability values of the prepared membranes were evaluated at a constant feeding pressure of 3 bar, effective area of the tested membrane of 16.38 cm^2^, and temperatures between 25 °C and 55 °C. The volumetric flow rate of CO_2_ and CH_4_ gases in permeate side was evaluated by a bubble flow-meter and pure gas permeability were calculated according to Equation (2) and all effective parameters are defined in Table 1, while the ideal CO_2_/CH_4_ selectivity (α) of the synthesized membranes was determined using Equation (3) [42]. To increase the results accuracy, the gas permeation experiments were repeated three times for each batch and the average values were listed.

(2) $$\mathrm{Pi}=10^{10}\;\times \;\frac{273.15}{76}\;\times \;\frac{p}{T}\;\times \;\frac{\mathrm{Qi}.l}{\Delta p.A}$$

(3) $$\alpha_{\mathrm{CO}2/\mathrm{CH}4}\;=\;\frac{\mathrm{PCO}2}{\mathrm{PCH}4}$$

## 3. Results and Discussion

### 3.1. Dispersion and Morphology of the Synthesized Membranes

**Figure 5:**
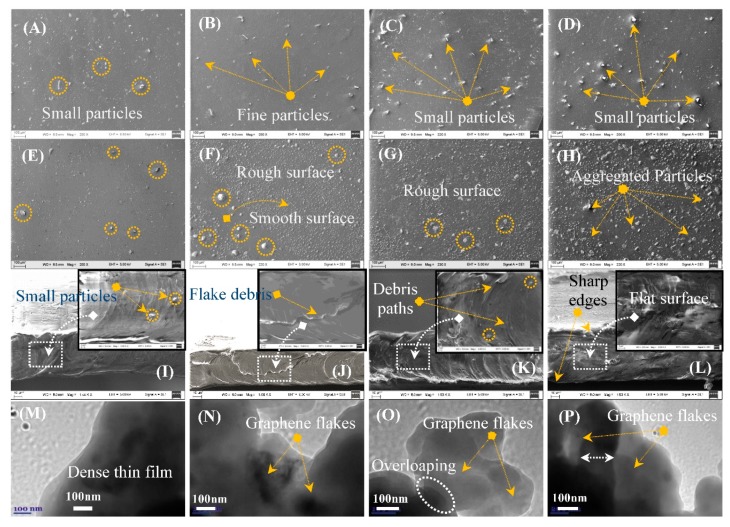
(**A**–**L**) SEM images and (**M**–**P**) TEM images.

Figure 5A–H shows the surface morphology of all the synthesized membranes using SEM examination at 100 μm after they have been cleaned in ethanol emulation using ultrasound. As shown in SEM images, the surface of pristine membrane (Figure 5A) contaminated by several debris in the form of small particles (indicated in the circles) was formed during solidification process and solvent evaporation caused the above defects. After adding PL (Figure 5B), the surface became smoother due to its modification [43]. Also, the number of particles decreased significantly and they became finer (indicated by arrows). After adding 0.05 and 0.1 wt.% GA to PL/PEBA matrix (Figure 5C,D), small particles started to appear again, especially at 0.1 wt.% as a result of poor dispersion. The amount of GA was not sufficient to cover all surface areas. At 0.2 and 0.3 wt. % of GA (Figure 5E,F), GA started to distribute uniformly and the surfaces became rough. However, few smooth surfaces appeared due to poor dispersion. At 0.4 wt.% of GA (Figure 5G), GA started to distribute uniformly and the surfaces became absolutely rough due to GA incorporated on the surface of the membranes, what leads to increased viscosity of the solution leading to increased surface tension and fast solidification [44,45]. When GA concentration reached the peak (Figure 5H), GA started to aggregate in the form of block particles which led to particle agglomeration owing to force interaction between GA particles.

Sine PEBA is classified as a very ductile material and this characteristic became even more evident after mixing PEPA with PL (highly plasticized) [38,43], it was hard to prepare fracture samples in liquid Nitrogen; therefore, sharp cutter was used to prepare fracture surfaces. Figure 5I–L shows the cross-section morphology of the synthesized membranes of pure PEBA and nanocomposite samples, particularly PEPA2 (poor dispersion) and PEPA4 (uniform dispersion). As shown in the SEM micrograph of the PEPA sample (Figure 5I), the fracture surface was completely deformed by numerous distortions and had many paths, the number of which was increasing with the addition of PL (Figure 5J). This means that the sample became plasticized and thus, in compliance with ductile failure. After addition of 0.4 wt.% of GA (Figure 5K), the fracture surface became brittle with small amount of debris of uniform thickness, which means that the surface became harder and in compliance with brittle failure [46]. Also, some smaller particles were still sticking out from the damaged surface (inside the yellow squares), which manifests ductile phenomenon (winding or spline surface). At high concentration, brittle fracture reappeared relatively smooth and the surface became flat, harder, and having sharp edges, manifesting brittle phenomenon (Figure 5L). 

Figure 5M–P shows surface microscopic morphology of all the synthesized membranes using TEM at 100 μm. As shown in the TEM images, the pure sample had a dense-thick structure, while the PEPA1 sample contained several thin GA flakes. By raising the concentration of GA up to 0.4%, the amount of these flakes increased significantly in vertical and horizontal plane overlapping a little between GA layers (indicated by white ellipse). When GA concentration was the highest, the overlapping increased significantly causing the aggregation phenomenon. The TEM results confirmed that GA flakes with 0.4 wt.% concentration were dispersed successfully uniformly in the PEPA matrix [35].

### 3.2. Chemical Composition of the Synthesized Membranes

**Figure 6:**
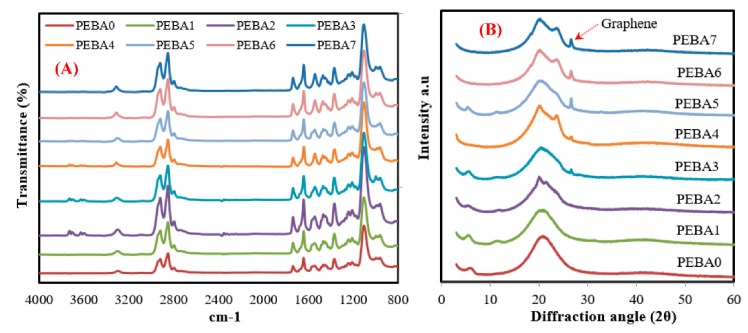
(**A**) FTIR and (**B**) XRD analysis of the synthesized membranes**.**

Figure 6A,B displays the FTIR spectra and XRD pattern of the pristine PEBA and composite membranes, respectively. FTIR results (Figure 6A) showed that the pristine sample exhibited several strong bands: 1092 cm^−1^ corresponding to the –C–O–C– group (Segment I), 1640, 1730, and 3270 cm^−1^ indicating the –HNCO–, O–C=O, and –NH– groups (Segment II), 2858 cm^−1^ representing –CH_2_– (Segment III), respectively. These existing segments corresponding to poly (ethylene oxide) (PEO), polyamide 6 (PA-6), and soft and hard segment in PEBA are considered to be the main components in PEGA. All the samples had the same peaks and groups [47]. All these groups did not alter in all the samples, even after having been mixed with PL. However, after mixing with GA, very weak peaks appeared at 3311 cm^−1^ and 1726 cm^−1^, corrosponding to hydroxyl and carbonyl groups of GA, respectively, which means that GA integrated with PEPA [39,44]. 

XRD pattern was used to confirm the FTIR results and to check crystallization peaks of the synthesized membranes. As mentioned before, PEPA is a semicrystalline copolymer that composed of crystal PA-6 and amorphous PEO segments. As displayed in XRD results (Figure 6B), an intensive crystallization peak notes in the XRD pattern of virgin PEBA in the scope from 14° to 27°, related to the hydrogen bonding between PA-6 chains (larger crystalline region) [48,49]. Also, no other characteristics were noted when PL was added to “PEBA1”, which means that crystallinity of virgin membrane was not affected by PL added to “PEBA1”. Interestingly, when GA was added, a single sharp peak belonging to GA was appeared at 26.4° and the intensity of this peak increased significantly by increasing the GA content. Also, PEBA nanocomposite membranes show reduced peak intensity of PA-6 segment with the increases of GA concentration, pointing to the smaller crystalline phase in the synthesized membranes [31]. In addition, incorporation of GA led to reduce crystallinity of these polymer segments by the disturbing arrangement of PA-6 chains, thereby providing a probability to improve gas permeation performance [50]. The presence of these peaks in XRD pattern of the synthesized membranes confirms that the developed approach helped strongly to distribute GA uniformly inside the matrix [35,51].

### 3.3. Thermal Properties of the Synthesized Membranes

**Figure 7:**
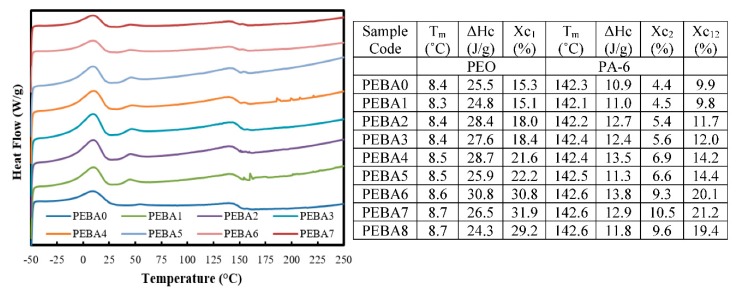
Typical DSC curves and calorimetric data of the synthesized membranes.

Figure 7 showed DSC curves and calorimetric data of the synthesized membranes and explored effect of GA and PL fillers on glass transition temperatures, melting temperature (Tm), and crystallinity (Xc) of the pristine membrane. As shown, DSC measurements contained two soft domains in the range from −39 to 29 °C for PEO part and in the range from 93 to 161 °C for PA-6 part, while melting enthalpy was in the range of 24.3–30.8 J/g for PEO fraction and in the range of 10.9–13.8 J/g for PA-6 fraction. Meanwhile, the melting temperature for each fraction was not affected significantly by adding PL and GA [52,53]. With regard to crystallinity degree (*Xc*), it was noted that the average crystallinity of PEO and PA-6 (*Xc_12_*) of the pristine membrane did not change by adding PL. This means that pristine membrane was prepared successfully by extrusion and casting process without any fusion defects [38] and these results compatible with XRD results. Also, it was noted that by adding GA to PL/PBPA membrane, *Xc_12_* increased significantly up to 0.4 wt. % of GA and thus improving by ~52% (from 9.9% to 21.2%) for both domains (PEO and PA-6). This increase due to GA resulted in high surface area to volume ratio, leading to fast interaction and better incorporating of GA with PEO and PA molecules. This led to reduced friction between PEBA chains and GA, meaning better bonding between their molecular structures, and thus improving the crystallinity degree of the obtained membranes [38]. 

**Figure 8:**
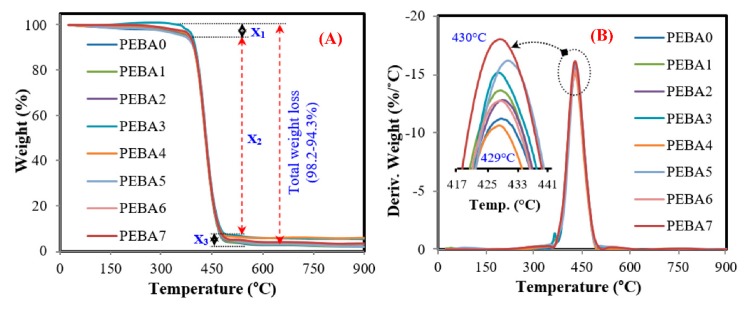
(**A**,**B**) typical TGA and DTG curve analysis of the synthesized membranes, respectively.

Figure 8 shows TGA and DTG results of the synthesized membranes, where PEBA showed one-stage decomposition corresponding to the random-chain disengage mechanism of the basic PEO and PA-6 chain located in the range (378–493 °C). As PL/PEBA and GA/PL/PEBA showed two-step decomposition, the first step represents PL decomposition in the range 218–377 °C with average weight loss of 2% (the exact amount added during the mixing process) [54], while the second decomposition is related to PEO and PA-6 in the range (378–493 °C). However, all samples had the same total weight loss, thus indicating that the presence of GA nanofiller does not affect significantly the thermal degradation behavior in the synthesized membranes; however, thermal stability in the form of mass loss was improved and this result is in agreement with results in the literature [41]. 

### 3.4. Mechanical Tensile Properties of the Synthesized Membranes

**Figure 9:**
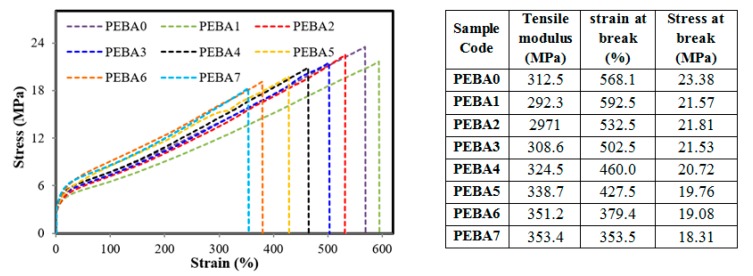
Stress-Strain curves of the synthesized membranes**.**

The stress-strain curves and mechanical properties of the synthesized membranes are presented in Figure 9. It seems that by adding PL, tensile strength and elasticity modulus were reduced by 7% and 6%, respectively, while the strain increased a little by 4% due to plasticity effect [55]. By adding GA to PL/PEBA samples, tensile strength and stain decreased when compared to a virgin sample, while the elasticity modulus increased and membranes became of more rigid and hard structure than the virgin PEBA membrane. This happened because mixing of GA with PEPA restricted the molecular rearrangement of polymer chains during the casting and solidification process [41]. The nanocomposite membranes manifested less changes in mechanical properties when compared to pure membrane because of good miscibility and higher interaction between GA nanofiller and PL/PEPA composite. Meanwhile, mechanical properties of membranes were enhanced and became more elastic, and the elastic modulus increased drastically.

### 3.5. Gas permeation Performance

**Figure 10:**
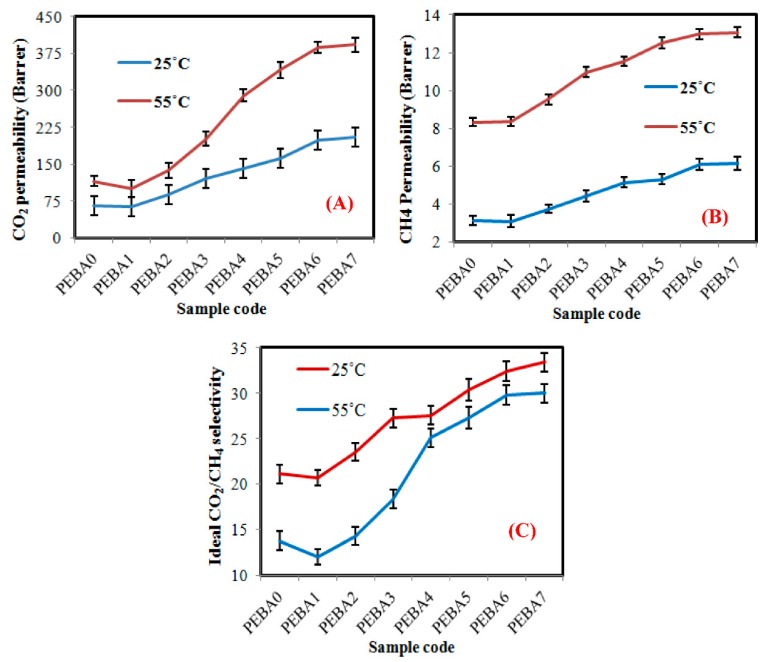
(**A**,**B**) CO_2_ and CH_4_ permeability and (**C**) Ideal CO_2_/CH_4_ selectivity of the synthesized membranes at 2 bar and various temperatures (25 and 55 °C).

Figure 10 illustrates the effect of PL adding and GA loading on the permeability of CO_2_ and CH_4_ gases and on the ideal CO_2_/CH_4_ selectivity of the synthesized membranes at 2 bar and various temperatures (25 and 55 °C). As shown in the figure, at the lowest temperature (25 °C), the permeability of CO_2_ and CH_4_ gases of the neat PEBA are estimated by 66 and 3.1 Barrer, respectively. These results agree with the literature, where polymer membranes, including PEBA are characterized by their high-free volume, which considers the main responsible for permeability [56]. Also, the amount and distribution of free volume in the substrate may influence the way of molecules pack together and its permeability [57]. In addition, it seems that permeability of the specified gases was not affected significantly by PL addition, as a result of the molecular structure of PEBA chain, which is composed of compact homochiral sheets. This form makes it difficult for molecules to pack and rearrange and fill space [17], even at the lowest concentration of GA (PEBA2). When GA loading was increased from 0.05 to 0.4 wt.%, permeability of CO_2_ and CH_4_ gases increased from 88.12 to 197.86 Barrer (increasing by ~56%) and from 3.74 to 6.10 (increasing by ~39%) Barrer, respectively (Figure 10A), where the presence of GA restricts the conformational freedom of PEBA chains in its vicinity, which may frustrate PEBA chain ability to group together and cause a curvature of the surface at the nanoscale [58], thus increments of the PEBA amorphous structure, fractional free volume and chain mobility as a result of decreasing in the crystallinity of the synthesized membranes (confirmed by the XRD and FTIR results), which causes a significant increase in CO_2_ permeability and a little in CH_4_ permeability of the synthesized membranes [59]. This can contribute to better affinity of GA to CO_2_ than CH_4_ and also to create selective voids at GA/PEBA interface, improving by 54% in the ideal CO_2_/CH_4_ selectivity when compared to pure membrane (Figure 10B). When the biggest GA amount was loaded (PEBA7), GA started to cluster together in the form of aggregated particles that the obstructed permeability of CO_2_, and CH_4_ gases inside the PEBA matrix [42]. These results agree with SEM and TEM results. At the highest temperature (55 °C), the permeability of CO_2_ and CH_4_ gases of the virgin and the nanocomposite membranes, increased almost similar to the membranes tested at a lower temperature. It is because synthesized membranes became more elastic under the applied temperature, which led to changing the dense structure into a fibrous structure, thus gases could pass smoothly, especially in case of polymer nanocomposite [60]. Also, the ideal CO_2_/CH_4_ selectivity at the high temperature was improved significantly from 21.10 (PEBA0) to 32.43 (PEBA7), improving by ~35%. 

As mentioned in the introduction section, recently, several types of polymer membranes have been developed for CO2/CH4 separation (e.g., PDMS, Pebax 1657, Pebax, PIM-1, Matrimid^®^, PSF, PI, SPEEK, PSF, etc.) [59]. Among the reviewed membranes, PDMS exhibited a lowest CO_2_/CH_4_ selectivity enhancement (3.2) [61], while Pebax 1657 gave a higher CO_2_/CH_4_ selectivity enhancement (9.3) [15]. For other polymer types, there exist conflicting results in CO_2_/CH_4_ selectivity. For example, the obtained results by Castro-Muñoz al. (2019) showed that the CO_2_/CH_4_ selectivity of Matrimid^®^ membrane is estimated by ~17 [62], while the obtained results by Abdollahi al. (2018) was ~31 [63]. Since Pebax membranes have a chemical structure and composition similar to PEBA with reasonable and accurate CO_2_/CH_4_ selectivity values, therefore Pebax was used in the present research for comparison. 

It is obvious that the results of the present work are very similar to the results listed in the literature, which reported that the adding of GA to Pebax membranes can be enhanced CO_2_/CH_4_ selectivity upto 28% [59]. According to the presented results, the suggested approach can be classified as a promising cost-quantitative technology for producing nanocomposite membranes with uniform dispersion and high ideal CO_2_/CH_4_ selectivity upto 32% and improved from 25 to 48% (based on the type of polymer and filler materials), when compared to results in the literature [59]. Also, the developed membranes are characterized by good CO_2_/CH_4_ selectivity stability even at high temperatures, which were estimated by 29% at 25 °C and 32% at 55 °C, which means that these membranes can be used in warm and cold ambient with the same selectivity performance. 

## 4. Conclusions

In the present research, the authors introduce novel Graphene/PEBA nanocomposite membranes that were first prepared to improve Carbon Dioxide permeability and CO_2_/CH_4_ selectivity. The extrusion process in presence of paraffin liquid was employed to produce GA/PEBA granules with uniform dispersion. Also, the crystallinity degree and fractional free volume of the fabricated GA/PEBA membranes were controlled preferentially, resulting in larger interchain spaces leads to achieve the better Carbon Dioxide permeability. The results show that only the addition of 0.4 wt.% of graphene could enhance the permeability of CO_2_ and CH_4_ gases effectively, which indicates great economic effects. The highest Carbon Dioxide permeability of 387 Barrer and CO_2_/CH_4_ selectivity of 32 was occurring at the optimal graphene loading of 0.4 wt.% at 55 °C. Finally, the developed membranes can provide a potentially suitable strategy to solve the limited application in boronate ester-linked graphene, and also it can be used to purify biogas obtained from pyrolysis and fermentation process.

## Figures and Tables

**Table 1:** Definition of the effective parameters of CO_2_ and CH_4_ permeability.

Parameter	Definition	Unit
Pi	CO_2_ or CH_4_ permeability	Barrer
T	Input temperature	K
p	Out pressure (Permeate)	cmHg
Δp	Pressure difference between input and output sides	cmHg
l	PEBA and composite membrane thickness	cm
A	PEBA and composite effective area	cm^2^
Qi	Volumetric flow rate of CO_2_ or CH_4_ gas	cm^3^s^−1^
